# A Real-World Evidence Study Assessing the Impact of Adding the Aerobika Oscillating Positive Expiratory Pressure Device to Standard of Care Upon Healthcare Resource Utilization and Costs in Post-Operative Patients

**DOI:** 10.1007/s41030-018-0055-9

**Published:** 2018-06-01

**Authors:** Chakkarin Burudpakdee, Aimee M. Near, Huan Huang, Dominic Coppolo, Vladimir Kushnarev, Jason Suggett

**Affiliations:** 1 IQVIA, Fairfax, VA USA; 2 Monaghan Medical Corporation, Syracuse, NY USA; 3 Trudell Medical International, London, ON Canada

**Keywords:** Aerobika, Cardiac, Healthcare cost, Healthcare resource utilization, Hospitalization, Incentive spirometry, Oscillating positive expiratory pressure, Post-operative, Thoracic, Upper abdominal

## Abstract

**Introduction:**

The aim of this real-world study was to measure the benefit of the Aerobika oscillating positive expiratory pressure (OPEP) device when added to standard of care (defined as incentive spirometry [IS]) for post-operative patients.

**Methods:**

Adults aged ≥ 18 years who were hospitalized for cardiac, thoracic or upper abdominal surgery between 1 September 2013 and 30 April 2017 were identified from IQVIA’s Hospital Charge Detail Master (CDM) database; the index date was the date of the first hospitalization for surgery. The control cohort (IS) included patients who had ≥ 1 CDM record within 12 months prior to the index date and ≥ 1 record after discharge, evidence of IS use during index hospitalization and no evidence of use of a PEP or OPEP device at any time during the study period. The Aerobika OPEP cohort was selected in a similar manner, except that patients were required to have evidence of Aerobika OPEP use during the index hospitalization. Aerobika OPEP patients were 1:1 matched to IS patients using propensity score (PS) matching. Hospital readmissions and costs were measured at 30 days post-discharge from the index hospitalization.

**Results:**

After PS matching, 144 patients were included in each cohort. At 30 days post-discharge, compared to the control (IS) cohort there were significantly fewer patients in the Aerobika OPEP cohort with ≥ 1 all-cause re-hospitalizations (13.9 vs. 22.9%; * p* = 0.042). The patients in the Aerobika OPEP cohort also had a shorter mean length of stay (± standard deviation) (1.25 ± 4.04 vs. 2.60 ± 8.24 days; *p* = 0.047) and lower total unadjusted mean all-cause cost per patient ($3670 ± $13,894 vs. $13,775 ± $84,238; *p* = 0.057). Adjusted analyses suggested that hospitalization costs were 80% lower for the Aerobika OPEP cohort versus the IS cohort (*p* = 0.001).

**Conclusion:**

Our results suggest that the addition of the Aerobika OPEP device to standard of care (IS) is beneficial in the post-operative setting.

**Funding:**

Trudell Medical International.

## Introduction

Post-operative pulmonary complications (PPCs) are a variety of conditions adversely affecting the respiratory system of the patient after anesthesia and surgery [1]. Examples of PPCs include atelectasis, pneumonia, pulmonary infiltrate, aspiration pneumonitis, pulmonary infection, exacerbation of chronic obstructive pulmonary disease (COPD), acute respiratory distress syndrome, acute respiratory failure and prolonged mechanical ventilation (longer than 48 h) [1]. The estimated incidence of PPCs varies greatly, ranging from as low as 5% to as high as 70%, depending on the type of surgery, which PPCs are included in the estimate and the study population [2]. PPCs are particularly common among patients who have undergone cardiac [3], thoracic [4] or upper abdominal surgery [5], and the patient’s underlying pulmonary status and comorbidities are just a few of the many factors which contribute to the individual’s risk of PPC after surgery [2].

PPCs are associated with a substantial clinical and economic burden to post-operative patients. They are associated with increased mortality in both the short and long term, with mortality in 30 and 90 days estimated to be 7- to 10-fold and 20-fold higher, respectively, among patients with a PPC compared to those without [1]. Similarly, PPCs are associated with increased healthcare resource use and costs. In particular, length of hospital stay has been shown to be prolonged by 13–17 days with the occurrence of a PPC [6]. This observed longer length of stay appears to be the largest driver of higher healthcare costs [7]. One retrospective study reported that the attributable cost of a PPC was over $52,000 per patient [8], and another study reported that PPCs were projected to add $3.43 billion in cost to the U.S. healthcare system [9]. These data highlight the economic burden of avoidable PPCs.

Strategies to prevent and treat PPCs include techniques of lung re-expansion using incentive spirometry (IS), which is the current standard of care [10]. Early research demonstrated that the use of IS decreases respiratory complications following major abdominal surgery [11]. However, the conclusions drawn by authors of recent systematic reviews are that there is a lack of evidence regarding the effectiveness of IS for the prevention of PPCs in patients who have undergone cardiac, thoracic or upper abdominal surgery [2, 12]. Specifically, one of the systematic reviews found that most studies showed no difference in patient outcomes (e.g. PPC, lung function) for patients using IS compared to controls [2].

Another commonly used intervention to prevent and treat PPCs is positive expiratory pressure (PEP) therapy, which involves breathing against expiratory resistance; PEP therapy can increase lung volume, reduce hyperinflation and improve airway clearance [13]. Oscillating positive expiratory pressure (OPEP) therapy is a variation of PEP that combines PEP with high-frequency vibrations or oscillations. It can thin and dislodge mucous by vibrating the airways during breathing, which assists in moving mucous up the airways [14]. In one clinical trial, the use of an OPEP device resulted in fewer cases of fever and shorter hospital stays among adults undergoing thoracic and upper abdominal surgery [15].

A variety of OPEP devices are currently available, including Aerobika (TMI, London, ON, Canada), which entered the U.S. market in September 2013. Clinical trial results have demonstrated that use of the Aerobika OPEP device significantly improves dyspnea, quality of life, exercise capacity and ease in bringing up sputum in patients with COPD and bronchiectasis [16]. In another clinical trial, the Aerobika OPEP device was further shown to significantly improve ventilation in patients with COPD [17]. In addition, real-world evidence suggests that the use of the Aerobika OPEP deviceamong COPD patients significantly reduces exacerbations by nearly 30% [18]. These data suggest that the use of the Aerobika OPEP device in the post-operative setting may help lower the incidence of PPCs, thereby reducing the risk of re-hospitalization and lowering the economic burden of PPCs in the post-operative population.

 The aim of this retrospective real-world study was to measure the benefit of the Aerobika OPEP device when added to standard of care (IS) for cardiac, thoracic and upper abdominal surgery patients.

## Methods

This retrospective database study utilized patient data stored in IQVIA’s proprietary Hospital Charge Detail Master (CDM) database pertaining to the period between 1 September 2012 and 31 May 2017. The CDM database manages daily transactional patient charges from over 650 hospitals from 46 states in the USA, covering 7 million annual inpatient stays and 60 million annual outpatient visits. Patient-level data include healthcare services from hospital departments (inpatient, outpatient clinic, emergency department, pharmacy) and encounters associated with ICD-9-CM/ICD-10 diagnosis codes and Current Procedural Terminology codes. Information on drugs and devices dispensed are available and reported in text fields in the database. Detailed charges associated with each visit are also available, as well as patient demographics and admission/discharge characteristics. In this database study, all patient-level data were anonymized and de-identified in compliance with the Health Insurance Portability and Accountability Act (HIPAA). As a retrospective cohort analysis of HIPAA-compliant de-identified patient data, no Institutional Review Board (IRB) review or clinical trial registration was required for this study.

Patients with a hospitalization related to a cardiac, thoracic or upper abdominal surgical procedure between 1 September 2013 and 30 April 2017 (i.e. the selection window) were selected into the study; the index date was defined as the first date of hospitalization with surgery. Patients were required to be at least 18 years of age and to have at least one record in the CDM database within 12 months prior to the index date (i.e. the baseline) and at least one record after the discharge date of the index hospitalization. All patients were required to have evidence of IS use during the index hospitalization. Patients were excluded if they had a prior cardiac, thoracic or upper abdominal surgery within 30 days of the index date, had more than one type of surgery during the index hospitalization or had incomplete demographic data (i.e. missing age, gender, payer type or geographic region).

Two patient cohorts were then developed. The first included patients for whom there was evidence of the use of the Aerobika OPEP device during the index hospitalization. These patients were also required to have no evidence of use of the Aerobika OPEP device at any time before the index date and no evidence of use of any other PEP or OPEP device at any time during the study period. An IS (control) group was also selected that consisted of patients for whom there was no evidence of the use of any PEP or OPEP device at any time during the study period.

Once the selection of the Aerobika OPEP cohort was complete, these patients were propensity score (PS) matched at a 1:1 ratio to IS patients, using the greedy nearest neighbor matching technique. PS matching used a logit regression constructed from patient characteristics measured during the 12-month period before the index date and during the index hospitalization. This methodology is commonly used in observational studies since it mimics the selection process of randomized clinical trials and decreases bias in the estimation of treatment effects between comparison groups. The following variables were included in the PS model: age category, gender, region, payer type, positive airway pressure device use during index hospitalization, surgical procedure, index year, Charlson Comorbidity Index (CCI) category, comorbid conditions (acute respiratory tract infections, asthma, atrial fibrillation, bronchiectasis, cardiovascular disease, COPD, congestive heart failure, malignancy, obstructive sleep apnea, obesity, pulmonary hypertension, pulmonary fibrosis, peripheral artery disease), medication history and medication use during index hospitalization (antibiotics, proton pump inhibitors, long-acting β2-agonist [LABA], long-acting muscarinic antagonist [LAMA], short-acting β2-agonist [SABA], short-acting muscarinic antagonist [SAMA], inhaled corticosteroids [ICS], oral corticosteroids, ICS/LABA combination, SABA/SAMA combination). In addition, preliminary analyses suggested significant heterogeneity between the Aerobika OPEP group and the IS group in terms of frequency of PPCs during the index hospitalization and the cost and length of stay of the index hospitalization. This is reflective of how the Aerobika OPEP device is currently used in clinical practice as a reactive intervention when a complication develops after surgery. Therefore, as well as the length of stay and cost of index hospitalization, the presence of each of the following PPCs during the index hospitalization (yes/no) was included in the PS model: respiratory failure, atelectasis, hypoxemia, pulmonary edema, pulmonary embolism, pleural effusion, pneumothorax, pulmonary eosinophilia, pneumonia, other pulmonary infection and tracheobronchitis.

Outcomes occurring within 30 days post-discharge of the index hospitalization were evaluated. These outcomes included the number and proportion of patients with at least one all-cause hospital readmission, total length of stay (days) of the rehospitalization, time to first hospital readmission (days) among patients who had at least one hospital readmission (conditional mean), number and proportion of patients with at least one hospital readmission with the procedure code indicating chest X-ray (proxy for respiratory complication) and total all-cause costs of hospital-related events. CDM data consists of charges for healthcare services; therefore, a cost-to-charge ratio of 0.4770 was used to convert charges reported in the CDM database to estimated costs. The ratio was calculated based on the average cost-to-charge ratios published by the Healthcare Cost and Utilization Project for the 2014 National Inpatient Sample. Costs were inflation-adjusted to 2017 U.S. dollars using the Medical Care component of the U.S. Consumer Price Index for All Urban Consumers [19].

Baseline patient characteristics were reported using descriptive statistics. The *p* value was assessed to evaluate balance of variables between Aerobika OPEP patients and matched IS patientsafter PS matching. For study outcomes, pair-wise comparisons were made between the Aerobika OPEP group and the IS group for each study measure. The nonparametric Wilcoxon signed-rank test was used to compare continuous variables, and the nonparametric McNemar/Bowker test was used to compare categorical variables. A *p* value of < 0.05 was consideredto be statistically significant for all study measures.

The additional effect of Aerobika OPEP (vs. IS alone) on total all-cause hospital costs post-discharge was evaluated using a multivariate generalized linear model (GLM) with a gamma distribution and a log link in the matched population. The following covariates were adjusted to control for remaining imbalance after PS matching: comorbid conditions (atrial fibrillation, diabetes, hypothyroidism, obesity, stroke or transient ischemic attack) and the log of total healthcare costs during index hospitalization. If two variables were highly correlated, the most clinically relevant variable was included in the model (e.g. oxygen use was excluded since CCI and PPC during the index hospitalization were included). All analyses were conducted using SAS version 9.4 (SAS Institute Inc., Cary, NC, USA).

## Results

**Fig. 1:**
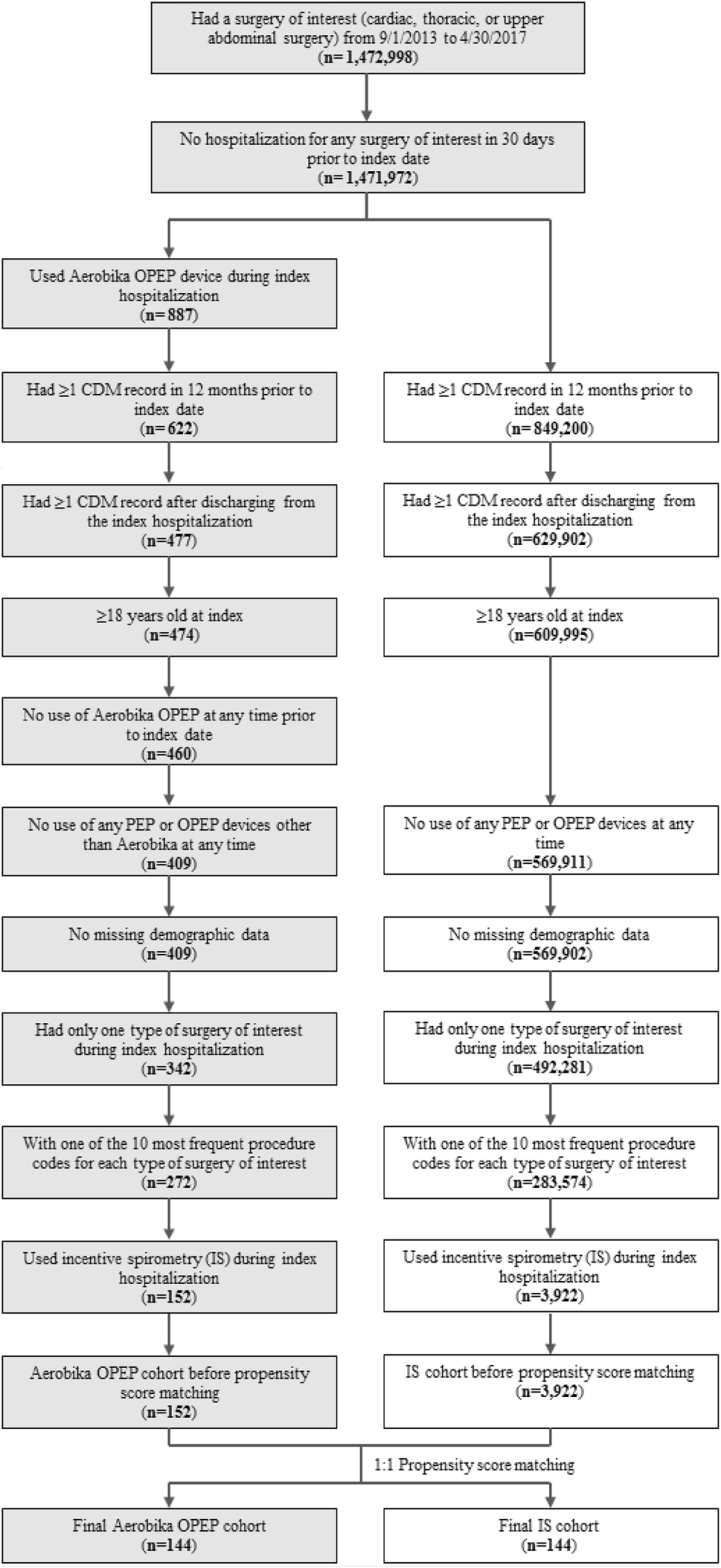
Flow diagram of patient selection.* CDM* Charge Detail Master database,* IS* incentive spirometry (standard of care),* OPEP* oscillating positive expiratory pressure,* PEP* positive expiratory pressure

A total of 887 cardiac, thoracic or upper abdominal surgery patients hospitalized between 1 September 2013 and 30 April 2017 were identified to have used the Aerobika OPEP device. After the selection criteria were applied, the study sample comprised 152 Aerobika OPEP patients and 3922 IS patients (Fig. 1). After PS matching, a total of 144 Aerobika OPEP users and 144 matched controls were identified; the matched cohorts were well balanced on all baseline characteristics (all *p* values > 0.05), except for evidence of chest X-ray during the 12-month pre-index period (*p* = 0.039; 59.0% Aerobika OPEP vs. 45.8% IS). The mean age of matched cohorts was around 65 years, with more than one-third of the patients in both cohorts aged 65–74 years (Table 1). A higher proportion of males (> 60%) was observed in both cohorts, and more than one-half of the patients were insured by Medicare Risk. The mean CCI score was 3.13–3.60, with more than one-third of the patients in both cohorts having a CCI score of ≥ 4. The most prevalent comorbid conditions were cardiovascular diseases (≥ 49.3%), COPD (≥ 40.3%), obesity (≥ 34.0%), anxiety/depression (≥ 33.3%), diabetes (≥ 33.3%) and malignancy (≥ 30.6%).

**Table 1:** Demographic and baseline clinical characteristics of the Aerobika OPEP and control cohorts

Measures	Aerobika OPEP device users (*N* = 144)	IS users (*N* = 144)	*p* value^a^
Age (years) at index			
Mean (SD)	65.4 (13.3)	65.9 (13.1)	0.672
Median (min, max)	67 (20, 85)	69 (24, 85)	
Age categories (years)			0.396
18–34	4 (2.8%)	5 (3.5%)	
35–50	16 (11.1%)	13 (9%)	
51–64	37 (25.7%)	36 (25%)	
65–74	52 (36.1%)	56 (38.9%)	
75+	35 (24.3%)	34 (23.6%)	
Gender			0.803
Female	55 (38.2%)	57 (39.6%)	
Male	89 (61.8%)	87 (60.4%)	
Payer type			0.969
Commercial	24 (16.7%)	21 (14.6%)	
Medicaid	4 (2.8%)	5 (3.5%)	
Medicare risk	73 (50.7%)	78 (54.2%)	
Self-insured	2 (1.4%)	4 (2.8%)	
Unknown	41 (28.5%)	36 (25%)	
Type of surgery during index hospitalization			0.963
Cardiac surgery	60 (41.7%)	57 (39.6%)	
Thoracic surgery	63 (43.8%)	65 (45.1%)	
Upper abdominal surgery	21 (14.6%)	22 (15.3%)	
Index year			0.674
2013	11 (7.6%)	16 (11.1%)	
2014	85 (59%)	80 (55.6%)	
2015	46 (31.9%)	45 (31.3%)	
2016	2 (1.4%)	3 (2.1%)	
2017	0 (0%)	0 (0%)	
Admission through emergency department	54 (37.5%)	64 (44.4%)	0.225
Receipt of chemotherapy (during baseline and index hospitalization)	17 (11.8%)	14 (9.7%)	0.578
Receipt of radiation therapy (during baseline and index hospitalization)	3 (2.1%)	1 (0.7%)	0.317
PAP use during index hospitalization			
Handheld	62 (43.1%)	64 (44.4%)	0.816
Ventilation^b^	38 (26.4%)	41 (28.5%)	0.668
Charlson comorbidity index (CCI)^c^			
Mean (SD)	3.1 (2.3)	3.6 (2.6)	0.139
Median (min, max)	3 (0, 11)	3 (0, 11)	
Categories			0.979
0	14 (9.7%)	14 (9.7%)	
1	22 (15.3%)	19 (13.2%)	
2	31 (21.5%)	24 (16.7%)	
3	23 (16.0%)	22 (15.3%)	
4+	54 (37.5%)	65 (45.1%)	
Comorbid conditions			
Acute respiratory tract infections	9 (6.3%)	7 (4.9%)	0.617
Anxiety/depression	48 (33.3%)	51 (35.4%)	0.696
Asthma	18 (12.5%)	18 (12.5%)	
Atrial fibrillation	43 (29.9%)	50 (34.7%)	0.370
Bronchiectasis	1 (0.7%)	2 (1.4%)	0.564
Cardiovascular diseases	78 (54.2%)	71 (49.3%)	0.392
Chronic obstructive pulmonary disease (COPD)	58 (40.3%)	60 (41.7%)	0.814
Congestive heart failure	39 (27.1%)	43 (29.9%)	0.593
Diabetes	48 (33.3%)	57 (39.6%)	0.279
Hypothyroidism	19 (13.2%)	27 (18.8%)	0.228
Malignancy	44 (30.6%)	46 (31.9%)	0.773
Osteoporosis	10 (6.9%)	10 (6.9%)	
Obstructive sleep apnea	23 (16.0%)	23 (16.0%)	
Obesity	49 (34.0%)	42 (29.2%)	0.378
Pulmonary hypertension	16 (11.1%)	15 (10.4%)	0.842
Pulmonary fibrosis	3 (2.1%)	3 (2.1%)	
Peripheral artery disease	27 (18.8%)	31 (21.5%)	0.505
Stroke or transient ischemic attack	20 (13.9%)	31 (21.5%)	0.086
Thrombocytopenia	7 (4.9%)	11 (7.6%)	0.285
Presence of proxy of PPCs during baseline			
Chest X-ray	85 (59.0%)	66 (45.8%)	0.039*
Mechanical ventilation	3 (2.1%)	1 (0.7%)	0.317
Oxygen therapy	6 (4.2%)	3 (2.1%)	0.317

Values in table are presented as a number (of patients) with the percentage in parenthesis, unless stated otherwise

*IS* Incentive spirometry (standard of care), *PAP* positive airway pressure, *PPC* post-operative pulmonary complications, *OPEP* oscillating positive expiratory pressure,* SD* standard deviation

^a^Nonparametric Wilcoxon signed-rank test and nonparametric McNemar/Bowker test were used to assess the measures of Aerobika OPEP vs. IS users. Asterisk indicates significant difference at *p* < 0.05

^b^Included use of continuous PAP devices or bi-level PAP devices

^c^CCI was calculated using ICD-9/10 or diagnosis-related group (DRG) codes in any place in the medical claims during the baseline and on the index date

The most common type of surgery during the index hospitalization was thoracic surgery (≥ 43.8%), followed by cardiac surgery (≥ 41.7%) and upper abdominal surgery (≥ 14.6%). More than one-half of the patient population had index hospitalizations in 2014 and over 30% in 2015, with a few patients selected from later years. Aerobika OPEP patients and matched controls were balanced in terms of medication history and use during the index hospitalization for antibiotics, anticoagulants other than warfarin (warfarin use was low), proton pump inhibitors and respiratory medications (Table 2). There was no evidence of the use of medications for either pulmonary hypertension or pulmonary fibrosis in this study population. The most commonly used medications in both groups appeared to be antibiotics (≥ 94.4%) and anticoagulants (≥ 83.3%). The most commonly used medication for respiratory disease was SABA (≥ 45.1%) followed by a SABA/SAMA combination (≥ 40.3%).

**Table 2:** Medication history at baseline and medication use during the index hospitalization

Measures	Aerobika OPEP device users (*N* = 144)	IS users (*N* = 144)	*p* value^a^
Use of medications			
Antibiotics	138 (95.8%)	136 (94.4%)	0.593
Anticoagulants^b^	120 (83.3%)	126 (87.5%)	0.317
Proton pump inhibitors	15 (10.4%)	20 (13.9%)	0.353
Medications for pulmonary hypertension	0 (0%)	0 (0%)	
Medications for pulmonary fibrosis	0 (0%)	0 (0%)	
Medications for respiratory diseases			
Long-acting β2-agonists (LABA)	0 (0%)	1 (0.7%)	
Long-acting muscarinic antagonists (LAMA)	0 (0%)	1 (0.7%)	
LAMA/LABA combination	0 (0%)	0 (0%)	
Short-acting β2-agonists (SABA)	69 (47.9%)	65 (45.1%)	0.612
Short-acting muscarinic antagonists (SAMA)	1 (0.7%)	4 (2.8%)	0.180
Inhaled corticosteroids (ICS)	1 (0.7%)	6 (4.2%)	0.059
Oral corticosteroids (OCS)	14 (9.7%)	24 (16.7%)	0.096
ICS/LABA combination	20 (13.9%)	21 (14.6%)	0.866
SABA/SAMA combination	58 (40.3%)	60 (41.7%)	0.800
Theophylline	0 (0%)	4 (2.8%)	
Phosphodiesterase type 4 inhibitors	0 (0%)	0 (0%)	
Respiratory monoclonal antibodies	0 (0%)	0 (0%)	
Anti-inflammatory drugs	0 (0%)	0 (0%)	

Values in table are presented as a number (of patients) with the percentage in parenthesis

^a^Nonparametric Wilcoxon signed-rank test and nonparametric McNemar/Bowker test were used to assess the measures of Aerobika OPEP device vs. IS users

^b^Counts for anticoagulants other than warfarin: one Aerobika OPEP device patient and 11 IS patients had evidence of warfarin use

Aerobika OPEP patients and matched controls were balanced in terms of presence of PPCs during the index hospitalization (all *p* values > 0.05) (Table 3). Respiratory failure was the most common PPC (≥ 31.3%), followed by pneumonia (≥ 25.0%), pleural effusion (≥ 17.4%) and atelectasis (≥ 14.6%). The majority of patients had evidence of a chest X-ray (proxy for respiratory complication) during the index hospitalization (95.5%), and approximately one-half of these patients received mechanical ventilation (≥ 47.2%). Use of oxygen therapy during the index hospitalization remained imbalanced after matching (81.9% for Aerobika OPEP cohort vs. 39.6% for IS cohort; *p* < 0.001). Length of stay and cost of the index hospitalization were balanced between Aerobika OPEP device users and matched controls (*p* ≥ 0.109).

**Table 3:** Presence of post-operative pulmonary complications, length of stay and cost during the index hospitalization

Measures	Aerobika OPEP device users (*N* = 144)	IS users (*N* = 144)	*p* value^a^
Presence of PPCs			
Aspiration pneumonitis	0 (0%)	1 (0.7%)	
Acute respiratory distress syndrome	0 (0%)	1 (0.7%)	
Respiratory failure	45 (31.3%)	50 (34.7%)	0.522
Atelectasis	21 (14.6%)	27 (18.8%)	0.317
Bronchospasm	0 (0%)	3 (2.1%)	
Hypoxemia	9 (6.3%)	7 (4.9%)	0.564
Pulmonary edema	2 (1.4%)	3 (2.1%)	0.655
Pulmonary embolism	2 (1.4%)	0 (0%)	
Pleural effusion	25 (17.4%)	34 (23.6%)	0.160
Pneumothorax	11 (7.6%)	12 (8.3%)	0.827
Interstitial emphysema	0 (0%)	2 (1.4%)	
Pulmonary eosinophilia	1 (0.7%)	1 (0.7%)	
Pneumonia	36 (25%)	43 (29.9%)	0.317
Other pulmonary infections^b^	13 (9%)	16 (11.1%)	0.564
Tracheobronchitis	1 (0.7%)	0 (0%)	
Proxy of PPCs			
Chest X-ray	139 (96.5%)	139 (96.5%)	
Mechanical ventilation	68 (47.2%)	79 (54.9%)	0.210
Oxygen therapy	118 (81.9%)	57 (39.6%)	< 0.001*
Length of stay (days)			
Mean (SD)	10.9 (6.8)	12.0 (8.3)	0.242
Median (min, max)	9 (2, 41)	9.5 (2, 62)	
Total all-cause cost ($)			
Mean (SD)	$46,770 ($26,797)	$53,013 ($32,273)	0.109
Median (min, max)	$44,719 ($8829, $171,033)	$51,114 ($6133, $225,909)	

Values in table are presented as a number with the percentage in parenthesis, unless stated otherwise

^a^Nonparametric Wilcoxon signed-rank test and nonparametric McNemar/Bowker test were used to the measures of Aerobika OPEP vs. IS users. Asterisk indicates significant difference at *p* < 0.05

^b^Included acute nasopharyngitis (common cold) and other pulmonary infections

At 30 days post-discharge, there were significantly fewer patients in the Aerobika OPEP cohort with at least one all-cause hospital readmission than in the IS cohort (13.9 vs. 22.9%; *p* = 0.042). Similarly, comparison of the distribution of the number of all-cause readmissions suggested more IS patients than Aerobika OPEP patients had one (17.4 vs. 11.1%) or two (5.6 vs. 2.8%) hospitalizations (both *p* = 0.061) (Table 4). Approximately 20% of IS patients had a readmission during which time at least one chest X-ray was performed (suggesting respiratory complication) compared to 11.1% of Aerobika OPEP patients (*p* = 0.037). On average, the mean length of stay (± standard deviation) during the post-discharge period was shorter for Aerobika OPEP device users than for IS users (1.25 ± 4.04 vs. 2.60 ± 8.24 days; *p* = 0.047). Time to first all-cause readmission was similar between the two cohorts.

**Table 4:** Outcomes within 30 days post-discharge

Measures	Aerobika OPEP device users (*N* = 144)	IS users (*N* = 144)	*p* value^a^
Number and proportion of patients with at least one all-cause rehospitalizations	20 (13.9%)	33 (22.9%)	0.042*
Number and proportion of patients with a procedure code for chest X-ray during rehospitalizations	16 (11.1%)	29 (20.1%)	0.037*
Number of all-cause rehospitalizations per patient			
Mean (SD)	0.17 (0.44)	0.28 (0.56)	0.038*
Median (min, max)	0 (0, 2)	0 (0, 2)	
Categories			0.061
0	124 (86.1%)	111 (77.1%)	
1	16 (11.1%)	25 (17.4%)	
2	4 (2.8%)	8 (5.6%)	
Total length of stay (days) of the rehospitalization (not including the index hospitalization)^b^			
Mean (SD)	1.25 (4.04)	2.60 (8.24)	0.047*
Median (min, max)	0 (0, 33)	0 (0, 81)	
Time to first all-cause rehospitalization among patients who had at least one all-cause rehospitalization (days)			
Mean (SD)	12.65 (7.68)	10.30 (8.32)	0.188
Median (min, max)	9 (3, 30)	8 (1, 30)	
Total all-cause costs of inpatient events (not including the index hospitalization)			
Mean (SD)	$3670 ($13,894)	$13,775 ($84,238)	0.057
Median (min, max)	$0 ($0, $130,896)	$0 ($0, $987,864)	

Values in table are presented as a number with the percentage in parenthesis, unless stated otherwise

^a^Nonparametric Wilcoxon signed-rank test and nonparametric McNemar/Bowker test were used to assess the measures of Aerobika OPEP vs. IS users. Asterisk indicates significant difference at *p* < 0.05

^b^The entire length of stay of a rehospitalizations was included in the analysis as long as the admission date was within 30 days following the discharge date of the index hospitalization

The total unadjusted mean all-cause cost per patient within 30 days post-discharge was lower for the Aerobika OPEP cohort than for the IS cohort ($3670 ± $13,894 vs. $13,775 ± $84,238; *p* = 0.057). After controlling for comorbidities and cost of the index hospitalization in the GLM model (to adjust for any remaining imbalance in the matched population), hospitalization costs within 30 days post-discharge were shown to be 80% lower for the Aerobika OPEP cohort versus the IS cohort (*p* = 0.001; Table 5).

**Table 5:** Multivariate regression on all-cause hospitalization cost within 30 days post-discharge

Parameters	Estimate	Exponentiated estimate	*p* value
Cohort (Aerobika vs. IS)	− 1.616	0.199	0.001
Comorbid conditions (each as a binary variable)			
Atrial fibrillation	0.899	2.458	0.025
Diabetes	0.143	1.153	0.711
Hypothyroidism	− 1.415	0.243	0.003
Obesity	− 0.532	0.588	0.171
Stroke or transient ischemic attack	− 1.448	0.235	0.003
Log of total all-cause healthcare cost of index hospitalization	0.613	1.845	0.036

## Discussion

This is the first real-world study to evaluate healthcare resource utilization and costs associated with use of the Aerobika OPEP device in combination with the current standard of care (IS) among post-operative cardiac, thoracic, and upper abdominal surgery patients. We found that, compared to patients who only received IS during the index hospitalization, patients who used the Aerobika OPEP device in addition receiving IS were less likely to have all-cause hospital readmissions, had a shorter length of stay during readmissions and, overall, had significantly lower, adjusted all-cause inpatient costs during the 30 days post-discharge. The mean length of stay for readmissions within the post-discharge window was 1.35 days shorter for the Aerobika OPEP group compared to the IS group, which is consistent with earlier clinical trial findings that post-operative use of an OPEP device resulted in significantly shorter hospital stays among patients undergoing thoracic and upper abdominal surgery [15]. Total unadjusted mean all-cause inpatient costs over the 30-day post-discharge period per patient was about $10,000 lower for the Aerobika OPEP group than for the IS group, although this result did not reach statistical significance. After adjusting for additional cofounders, our regression model further confirmed this observed trend towards lower costs and reached statistical significance. Costs at 30 days are particularly relevant as providers and insurers continue to seek ways to reduce early re-hospitalizations in their patient population, and the Aerobika OPEP device is an inexpensive drug-free intervention that can prevent PPCs and reduce associated hospital costs.

Although not in the post-operative care setting, other real-world studies on the use of the Aerobika OPEP device in COPD patients suggest that the device improves outcomes by helping to open airways, mobilize and clear mucus and enhance drug deposition. One study by Burudpakdee et al. on patients with COPD found that the Aerobika OPEP device was associated with reduced visits to the emergency department, reduced number of hospital readmissions and reduced costs [18]. Another study found that the Aerobika OPEP device resulted in a direct medical cost savings of $553 per patient [20]. These data suggest that the reductions in healthcare resource use and costs associated with the Aerobika OPEP device among post-operative patients in our study may result from the beneficial effects of this device on improving airway structure and function [16, 17], thus reducing the risk of developing a PPC that warrants rehospitalization.

The results of this study suggest that the Aerobika OPEP device can improve patient outcomes, reduce early rehospitalizations and lower inpatient costs when used reactively in a post-operative population after the development of a complication. Based on our findings and taking into consideration both the low acquisition cost and clean safety profile of the Aerobika OPEP device, we suggest that the Aerobika OPEP device may be beneficial when used as standard of care in all post-operative patients. However, future studies are warranted to measure the benefits of Aerobika OPEP device use in all patients who undergo a surgical procedure to prevent PPCs.

This study has several limitations inherent to retrospective studies. First, our results can only establish associations and cannot identify cause-and-effect relationships. Second, the CDM data are primarily used for billing purposes and therefore are subject to potential coding errors. Moreover, they cannot provide as much clinical detail and accuracy as medical records. Relevant laboratory values and patient vitals are not available in the database; therefore, the severity of the condition was inferred using diagnosis codes as markers of disease severity, such as medication history, comorbidities and CCI score. Furthermore, these data comprise hospital records and study measures only reflective of care received in the hospital setting. Medications dispensed in community and retail pharmacies are underreported in this study. Office visits and care provided by primary care physicians and other physicians outside of the hospital setting are not captured in CDM and, therefore, our findings reflect the hospital setting only. Additionally, because CDM is an open database covering hospitals from 46 states in the USA, it is possible that not all inpatient events are captured in cases where a patient visits a hospital that does not contribute to the CDM database. However, the potential for underreporting inpatient events was minimized by requiring at least one CDM record both before and after the index date to ensure that patients were seeking care within the healthcare network captured in the CDM. Lastly, in this study, it is the addition ofthe Aerobika OPEP device to standard treatment (IS) that may be responsible for the improvements observed. These results may not be generalizable to a hospital where the treatment protocol does not require the use of IS as the standard of care, as clinical guidelines suggest IS is only recommended in certain cases [21].

Despite these limitations, the CDM hospital database is well-suited for this analysis of the use of the Aerobika OPEP device in the post-operative care setting. Treatment of severe post-surgery complications requires a visit to the emergency department or hospitalization, and the CDM database captures these types of events well since data are derived from the hospital setting. More importantly, a database study of Aerobika OPEP patients is only possible in the CDM dataset (as opposed to standard adjudicated claims) due to the lack of specific code for the Aerobika OPEP device and the need to identify patients using the device named in the billing description. Additionally, our study utilized a PS-matched cohort study design to minimize bias and regression analyses to control for potential confounders. The trend in lower all-cause inpatient costs was confirmed in our regression analysis, further strengthening the internal validity of our findings.

## Conclusions

This is the first real-world study to measure the benefit of the Aerobika OPEP device when added to the standard of care (IS) in the post-operative care setting. These findings suggest that the Aerobika OPEP device is associated with significantly fewer re-hospitalizations within 30 days post-discharge, shorter length of stay and lower costs associated with inpatient events compared to the standard of care (IS alone). Patients who utilized the Aerobika OPEP device incurred lower healthcare costs as a result of fewer complications warranting returns to the hospital and, therefore, it can be concluded that the Aerobika OPEP device may be an effective treatment for the management of post-surgery patients.
